# Urinary iodine concentration (UIC) could be a promising biomarker for predicting goiter among school-age children: A systematic review and meta-analysis

**DOI:** 10.1371/journal.pone.0174095

**Published:** 2017-03-22

**Authors:** Linlin Xiu, Gansheng Zhong, Xueman Ma

**Affiliations:** School of Basic Medical Science, Beijing University of Chinese Medicine, Beijing, China; Oklahoma State University, UNITED STATES

## Abstract

**Objectives:**

To evaluate whether urinary iodine concentration (UIC) can predict goiter among school-age children, and to assess the association between UIC and goiter prevalence.

**Methods:**

We searched the MEDLINE, EMBASE, Cochrane Library (Cochrane Database of Systematic Reviews), Web of Science, CNKI, VIP, and Wan Fang databases for relevant reports in both English and Chinese up to August 25, 2016. The mean differences (MD) and 95% confidence intervals (CI) were calculated for the UIC and goiter prevalence assessments. Pooled odds ratios and 95% CIs were used to compare the prevalences of goiter in the different UIC groups.

**Results:**

We identified 11 case-control studies, and found that children with goiter had lower UIC values, compared to children without goiter (MD: –1.82, 95% CI: –3.24, –0.40, p < 0.05). An increased risk of goiter was associated with UIC values of < 20 μg/L or > 200 μg/L.

**Conclusion:**

The results of our meta-analysis suggest that lower UIC values were associated with an increased risk of goiter, and that iodine deficiency may lead to an increased risk of goiter. Furthermore, we observed U-shaped relationships between UIC and the prevalence of goiter, which suggests that both severe iodine deficiency and excessive iodine intake may lead to increased risks of goiter.

## Introduction

Goiter remains an important public health concern, especially in developing countries [1–3]. During recent decades, goiter was mainly considered the result of inadequate iodine intake, although excessive iodine intake can also cause goiter, especially in areas with high iodine levels, such as coastal areas, areas with high iodine levels in the drinking water, and areas with poor iodine monitoring [4–6]. Thus, both inadequate and excessive iodine intakes are considered risk factors for goiter [7–10].

The measurement of urinary iodine concentration (UIC) in casual urine specimens is recommended for monitoring iodine status [1]. In addition, UIC is highly sensitive to recent changes in iodine intake, as up to 90% of iodine is absorbed and excreted in the urine [11]. Although iodine intake is a primary determinant of goiter formation [1], measuring UIC does not directly assess thyroid function and size. Nevertheless, excessively high or low UIC values in a population predict a high risk of goiter formation. The World Health Organization (WHO)’s classifications of iodine nutrition status are based on UIC values of <20 μg/L (severe iodine deficiency), 20–49 μg/L (moderate iodine deficiency), 50–99 μg/L (mild iodine deficiency), 100–199 μg/L (adequate iodine intake), 200–299 μg/L (more than adequate iodine intake), and >300 μg/L (excessive iodine intake). UIC levels of >300 μg/L are also associated with risks of iodine-induced goiter, hyperthyroidism, and hypothyroidism. School-age children (6–18 years old) are generally considered appropriate for assessing population-level iodine status, because they are readily accessible and susceptible to both iodine deficiency and excess [1].

As goiter formation reflects chronic iodine deficiency or excess, it can be used as a baseline assessment of a region’s iodine status [12], and goiter formation can be predicted and prevented by ensuring that the population has an appropriate and sustainable intake of iodine. In this context, UIC measurements are often used to evaluate the iodine nutritional status of a population [1], and can be used to track iodine status changes over time. Furthermore, spot urine specimens are easy to obtain, and urinary iodine assays are simple to understand and use. Thus, modern methods have made it feasible to process large numbers of samples at a low cost and to characterize the population-level distribution according to different cut-off points and intervals. Therefore, UIC is a convenient, inexpensive, and promising biomarker for predicting and preventing goiter in areas where goiter is endemic and long-term monitoring is warranted.

The present meta-analysis aimed to evaluate the evidence regarding whether UIC could predict goiter among school-age children, and to assess the goiter prevalences in different UIC groups.

## Methods

### Search protocol

The present meta-analysis was performed according to the PRISMA guidelines [13] and the Meta-analysis of Observational Studies in Epidemiology guidelines [14]. This meta-analysis was also registered in the PROSPERO registry (CRD42016043222). The systematic literature search was performed using the PubMed, EMBASE, Cochrane Library, Web of Science, Chinese Science and Technology Journal Database, China National Knowledge Infrastructure, and Wanfang databases to identify relevant reports in English and Chinese up to August 25, 2016. All databases were searched using the following key words urinary iodine, goiter and children. The detailed search strategy for PubMed was: (goiter [Title/Abstract] AND urinary iodine [Title/Abstract]) AND children [Title/Abstract] AND "humans" [MeSH Terms]. The reference lists of the returned articles were manually examined to identify any additional relevant studies.

### Selection criteria

The inclusion criteria were studies that: (a) assessed and reported UIC among school-age children with and without goiter, (b) compared the prevalences of goiter in different UIC groups based on the WHO categories, (c) used a case-control or cohort design, and (d) evaluated school-age children (6–18 years old). We excluded studies that did not fulfill the inclusion criteria, and also excluded conference abstracts and animal experimental studies.

### Data extraction

The following data were extracted from the included studies: (a) first author, (b) publication and study year, (c) study location, (d) study design, (e) sample size and number of goiter cases, (f) UIC values from children with and without goiter, and (g) the prevalences of goiter in the different UIC groups (<20 μg/L vs. >20 μg/L, <50 μg/L vs. >50 μg/L, <100 μg/L vs. >100 μg/L, <200 μg/L vs. >200 μg/L, and <300 μg/L vs. >300 μg/L). The extracted data were entered into an Excel file and Review Manager software by two of the authors.

### Study quality assessment

The quality of the included studies was assessed using the Newcastle-Ottawa Scale [15].

### Statistical analysis

Meta-analyses were used to provide overall estimates of UIC among children with and without goiter, and to compare the prevalences of goiter in the different UIC groups. The mean differences (MD) and 95% confidence intervals (CI) were calculated for the UIC and goiter prevalence data. The assumption of heterogeneity was assessed, and heterogeneity was considered present at a p-value of <0.1. To compare the prevalences of goiter in the different UIC groups, we calculated the pooled odds ratios (ORs) and 95% CIs. All analyses were performed using Review Manager software (version 5.3), and differences were considered statistically significant at p-values of <0.05.

## Results

### The included studies

**Fig 1:**
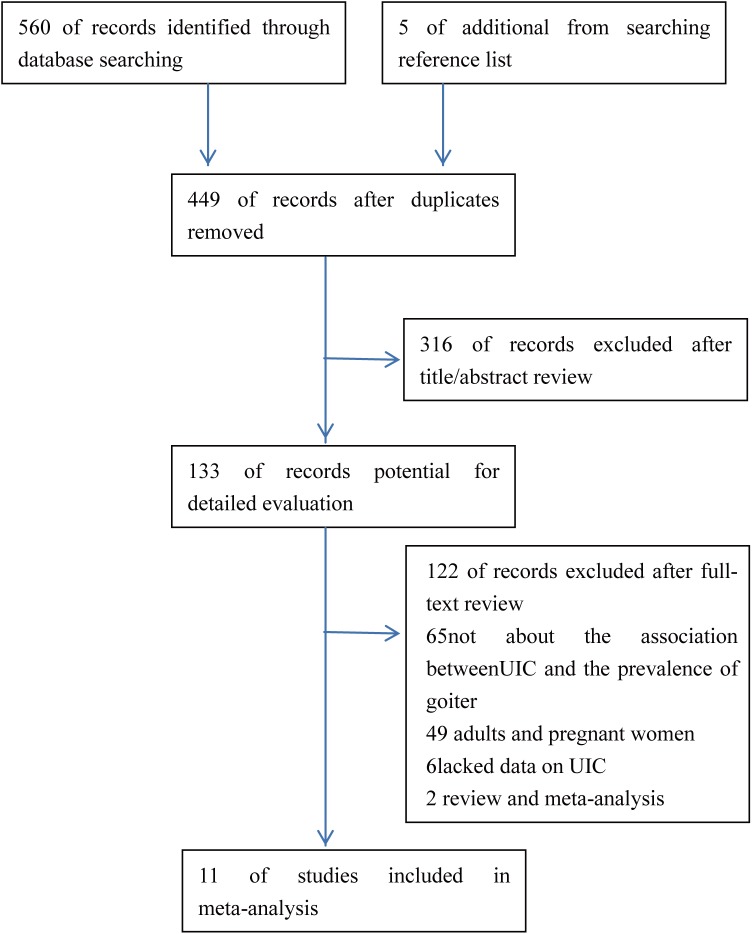
Flow diagram of the study selection process.

Our literature search identified 560 potentially relevant reports that were published up to August 25, 2016, and we also identified five other studies from the reports’ reference lists. However, we excluded 116 reports because of duplication, 316 reports because they were unrelated to the aim of this meta-analysis, and 122 reports for failing to fulfill the inclusion criteria after a review of the full text (primarily because they failed to compare UIC values between children with and without goiter). A total of 11 studies fulfilled our inclusion criteria and were included in the meta-analysis [16–26]. The selection process and outcomes are shown in Fig 1.

The basic characteristics of the 11 included studies are shown in Table 1. The studies were performed in Iran (n = 4), Turkey (n = 4), India (n = 1), and China (n = 1). All of the studies were cross-sectional case-control studies, and the numbers of participants ranged from 250 to 52,087. The study-specific quality assessment scores for the 11 studies are summarized in Table 1, and we defined high-quality studies as having quality scores of ≥6 (S2 Table).

**Table 1:** Characteristic of the included studies.

Study (year)	Country	Study design	Sample size	Cases/controls	NOS score	Outcome assessment
**Rezvanfar et al. [16] 2007**	Iran	Case-control	6,520	179/140	9	UIC in children with and without goiter
**Sethy et al. [17] 2007**	India	Case-control	1,248	95/346	8	UIC in children with and without goiter; goiter prevalences according to iodine status
**Cetin et al. [18] 2006**	Turkey	Case-control	500	152/348	8	UIC in children with and without goiter
**Cinaz et al. [19] 2004**	Turkey	Case-control	905	107/165	8	UIC in children with and without goiter
**Dodd et al. [20] 1993**	India	Case-control	866	489/377	8	UIC in children with and without goiter
**Özkan et al. [21] 2004**	Turkey	Case-control	250	119/131	8	UIC in children with and without goiter
**Sanjari et al. [22] 2014**	Iran	Case-control	5,380	130/40	8	UIC in children with and without goiter
**Liu et al. [23] 2010**	China	Case-control	52,087		8	Goiter prevalences according to iodine status
**Azizi et al. [24] 2008**	Iran	Case-control	6,000		8	Goiter prevalences according to iodine status
**Aminorroaya et al. [25] 2001**	Iran	Case-control	3,791		8	Goiter prevalences according to iodine status
**Egri et al. [26] 2006**	Turkey	Case-control	9,412	152/416	7	UIC in children with and without goiter; goiter prevalences according to iodine status

Eight studies [16–22, 26] reported information regarding UIC in children with and without goiter, and five studies [17, 23–26] investigated goiter prevalences according to iodine status.

### Heterogeneity

**Fig 2:**
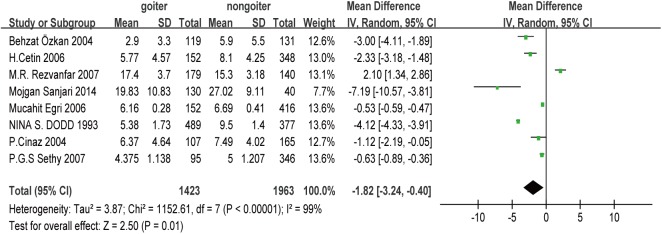
Forest plot showing the comparison of UIC between children with and without goiter.

As shown in Fig 2, our heterogeneity test provided a p-value of <0.05 and an I^2^ value of 99%, which confirmed that significant heterogeneity was present. Possible explanations for the heterogeneity include the studies evaluating different races and ethnicities, the different sample sizes, differences in the laboratory tests for determining UIC, and the possible effects of regional economic and cultural differences.

### Meta-analysis

#### Comparing UIC values from children with and without goiter

The pooled results from the comparison of UIC between children with and without goiter are shown in Fig 2. A total of 3,386 children were included in the analysis (1,423 cases with goiter and 1,963 cases without goiter). Children with goiter had significantly lower UIC values, compared to children without goiter (MD: –1.82; 95% CI: –3.24, –0.40; p < 0.05), and significant heterogeneity was observed (I^2^ = 99%; p < 0.05).

#### Association between iodine status and goiter prevalence

**Fig 3:**
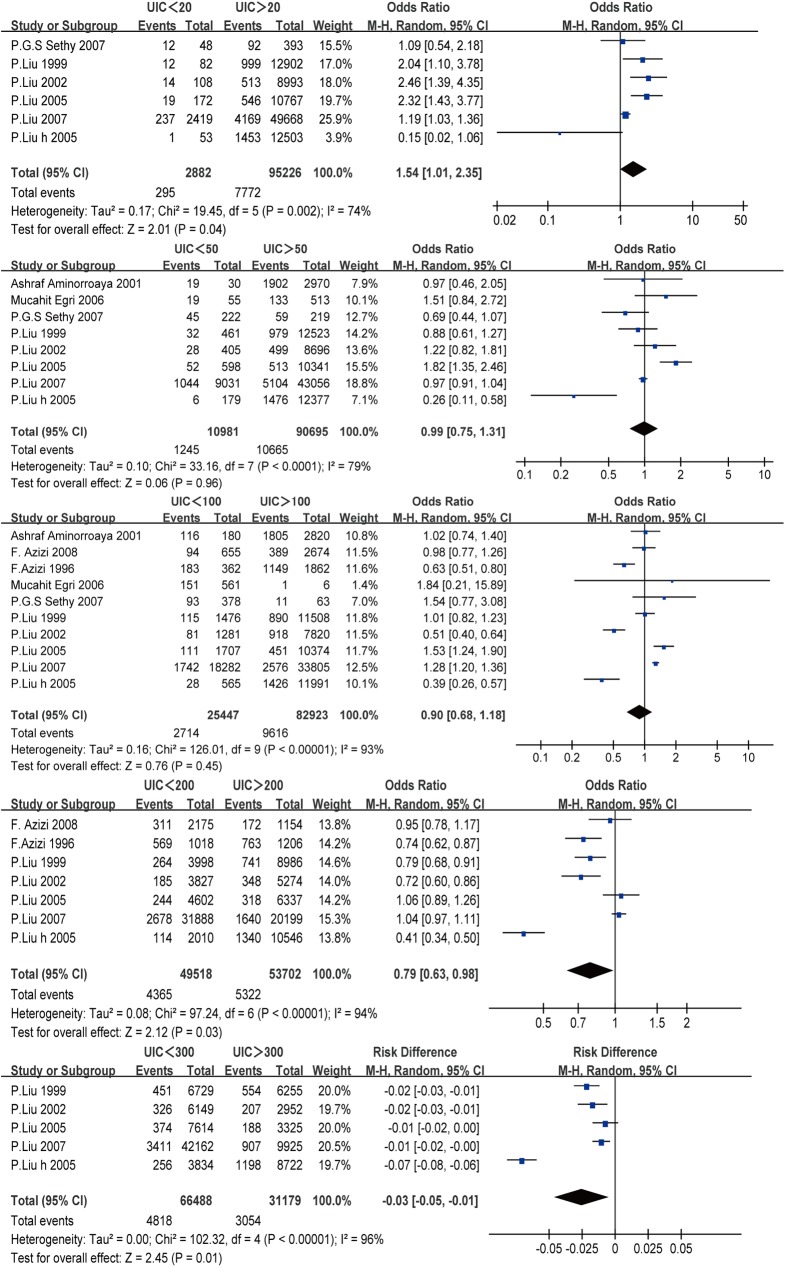
Forest plot showing the comparison of goiter prevalence in different UIC categories.

Fig 3 shows the association between iodine status and goiter prevalence, based on the different UIC categories.

We observed a U-shaped association between the UIC values and the prevalence of goiter. The pooled results for these comparison revealed increasing risks of goiter at both high UIC values (>200 μg/L) and low UIC values (<20 μg/L): <20 μg/L vs. >20 μg/L (OR: 1.54, 95% CI: 1.01, 2.35; p = 0.04), <50 μg/L vs. >50 μg/L (OR: 0.99, 95% CI: 0.75, 1.31; p = 0.96), <100 μg/L vs. >100 μg/L (OR: 0.90, 95% CI: 0.68, 1.18; p = 0.45), <200 μg/L vs. >200 μg/L (OR: 0.79, 95% CI: 0.63, 0.98; p = 0.04), and <300 μg/L vs. >300 μg/L (OR: –0.03, 95% CI: –0.05, –0.01; p = 0.01).

## Discussion

Iodine is an essential micronutrient that is required to synthesize thyroid hormones, and universal salt iodization is considered an appropriate method for iodine fortification in developed countries [27]. However, adverse effects have been recognized and careful monitoring is essential, as excessive iodine intake is associated with an increased risk of goiter and other iodine-related thyroid diseases. Thus, it is important to understand the association between iodine status and goiter prevalence after the implementation of the universal salt iodization. As UIC is highly sensitive to recent changes in iodine intake, UIC may be a reliable indicator for assessing, monitoring, and evaluating iodine status [28, 29]. For example, UIC might be a useful indicator of the effect of universal salt iodization and may help predict the occurrence of goiter.

We evaluated 11 case-control studies in the present meta-analysis, and found that children with goiter had significantly lower UIC values, compared to children without goiter, which indicates that lower UIC values were associated with an increased risk of goiter. Furthermore, as UIC is a reliable indicator for assessing population-level iodine status, it appears that iodine deficiency may lead to an increased risk of goiter. Similarly, an epidemical study has suggested that approximately 2.2 billion people live in areas with iodine deficiency and 30–70% of these people have goiter [30]. Although iodine deficiency is usually managed after the introduction of universal salt iodization, iodine excess has recently emerged as a noticeable public health issue. For example, several epidemiological studies have indicated that iodine excess is associated with iodine-induced hyperthyroidism and nodular goiters [22, 23, 31].

Based on the strong relationship between iodine status and goiter formation, it is important to evaluate the relationship between iodine status and goiter prevalence. Thus, we compared the prevalences of goiter among the different UIC groups based on the WHO classifications. This analysis revealed a U-shaped association between UIC and goiter prevalence, with higher prevalences observed at UIC values of <20 μg/L and >200 μg/L. Therefore, it appears that both severe iodine deficiency and excessive iodine intake may increase the risk of goiter, and this finding is consistent with the WHO recommended range for adequate iodine intake (100–199 μg/L).

Our meta-analysis has several strengths. First, most of the previous studies have focused on adults, rather than children. However, children are more sensitive to iodine deficiency or excess, and goiter or thyroid nodules are more frequently malignant during childhood, compared to during adulthood (26% vs. 5–10%, respectively) [32, 33]. Second, to the best of our knowledge, ours is the first study to evaluate the relationship between iodine status and goiter prevalence using a meta-analysis. The findings improve our understanding of how iodine status affects goiter prevalence, and suggest that UIC could be a promising biomarker for predicting goiter among school children, which could facilitate interventions to address iodine excess or deficiency. Third, our meta-analysis evaluated children from various races and locations, and the studies generally used long follow-ups, case-control designs, and large sample sizes. Thus, our results are unlikely to have been excessively influenced by the result from a single study.

Our meta-analysis also has several limitations. First, we did not have access to thyroid volume data, although thyroid gland inspection and palpation has been endorsed as part of the WHO criteria [1]. Thus, we were unable to evaluate the association between UIC and thyroid volume, which might have revealed the extent of the iodine status’ influence on goiter formation. Second, we did not have access to goiter classification data, and we were unable to evaluate the associations between iodine status and the different types of goiter. Third, we were unable to identify cases of autoimmune-related goiter, which might have distorted the association between UIC and goiter prevalence in our analyses. Fourth, although UIC reflects recent iodine status, it can also exhibit intra-day variations as a result of differences in iodine intake, which could have introduced information bias. Fifth, we were only able to evaluate reports in English and Chinese, and it is possible that reports in other languages or with negative results might not have been published and included in the databases that we searched. Thus, there is a possibility of both selection and publication bias.

## Supporting information

S1 Table The PRISMA check list. (DOC)

S2 Table The Newcastle-Ottawa Scale for assessing the methodological quality of the included studies. (XLS)
