# Correction: Urinary iodine concentration (UIC) could be a promising biomarker for predicting goiter among school-age children: A systematic review and meta-analysis

**DOI:** 10.1371/journal.pone.0181286

**Published:** 2017-07-07

**Authors:** Linlin Xiu, Gansheng Zhong, Xueman Ma

The Funding section is incorrect. The correct funding information is as follows: This study was supported by the National Natural Science Foundation of China (81573630).
